# Understanding the Links Between Perceiving Gratitude and Romantic Relationship Satisfaction Using an Accuracy and Bias Framework

**DOI:** 10.1177/19485506221137958

**Published:** 2022-12-02

**Authors:** Hasagani Tissera, Mariko L. Visserman, Emily A. Impett, Amy Muise, John E. Lydon

**Affiliations:** 1 McGill University, Montreal, Québec, Canada; 2 University of Toronto, Mississauga, Ontario, Canada; 3 York University, Toronto, Ontario, Canada

**Keywords:** gratitude, accuracy, bias, truth and bias model of judgment, relationship satisfaction

## Abstract

Perceiving a partner’s gratitude has several benefits for romantic relationships. We aimed to better understand these associations by decomposing perceptions into accuracy and bias. Specifically, we examined whether accuracy and bias in perceiving a partner’s experience (Study 1: *N_dyads_=* 205) and expression (Study 2: *N_dyads_=* 309) of gratitude were associated with romantic relationship satisfaction. Using the Truth and Bias Model of Judgment, we found that perceivers generally underestimated their partner’s gratitude, and lower perceptions of gratitude were related to lower perceiver satisfaction. Perceivers reported greater satisfaction when they assumed their partner’s gratitude was similar to their own. Partners reported greater satisfaction when perceivers accurately gauged their partners’ gratitude experience (but not expression) and lower satisfaction when perceivers underestimated their gratitude expression (but not experience). Overall, by decomposing gratitude perceptions into accuracy and bias, we provide insight into how these components differentially relate to relationship satisfaction.

Psychologists have long been interested in the topics of accuracy and bias of social perceptions. Research has revealed many cognitive biases that infiltrate our perceptions of the social world (Kahneman & Tversky, 1972; Murray et al., 1996; Tversky & Kahneman, 1973, 1974) and yet has also shown that our impressions are rooted in reality (Cronbach, 1955; Kenny & Albright, 1987; Swann & Gill, 1997). Although these topics were often studied in isolation, it appears that people can be both accurate and biased. This seeming contradiction has been discussed theoretically (Fletcher & Kerr, 2010; Funder, 1987; Gagné & Lydon, 2004; Kenny & Aceitelli, 2001) and, more recently, tested empirically (Biesanz, 2010, 2021; Edwards, 2002; West & Kenny, 2011), especially in the context of close relationships (e.g., Clark et al., 2017; Elsaadawy et al., 2021; Luo & Snider, 2009; Muise et al., 2016; Neff & Karney, 2005; Overall et al., 2012, 2015; Overall & Hammond, 2013; Peters & Overall, 2020; Spielmann et al., 2020; Visserman et al., 2019, 2021). The fast-growing nature of this literature is a testament to its high importance and interest. The present work advances this body of work by examining accuracy and bias in a novel domain: perceiving a romantic partner’s gratitude and its implications for relationship well-being.

## Gratitude

Gratitude is a positive, social emotion that arises when individuals appreciate the benefits of another person’s intentional actions toward them (McCullough et al., 2001). Aligning with the few studies that have explicitly examined perceptions of gratitude (e.g., A. M. Gordon et al., 2012; Schrage et al., 2022), past research employing dyadic designs indicates that one’s gratitude is related to various positive relational outcomes for their partner (e.g., Algoe et al., 2010, 2013; C. L. Gordon et al., 2011; Park, Johnson, et al., 2019; Schrage et al., 2022; Visserman et al., 2019), implying that *perceiving* others’ gratitude toward the self is associated with positive social outcomes for the perceiver. However, no prior work has examined the partner effects of perceiving gratitude (i.e., a partner’s outcomes in response to whether and how their gratitude is recognized). This is an important question to address as many relational processes are interdependent in nature (Kelley & Thibaut, 1978; Wieselquist et al., 1999). Moreover, past work has not revealed whether positive outcomes for perceivers are primarily driven by the accuracy or bias in perceiving a partner’s gratitude, or both. For example, people might benefit from seeing their partner’s relative levels of gratitude, as indexed as a high correlation between perceiver judgments and their partners’ actual gratitude, and/or they may benefit from displaying a directional bias, as indexed by an overestimation or underestimation of a partner’s gratitude. Decomposing perceptions into accuracy and bias can help identify which component is primarily driving the positive outcomes. As such, using a dyadic design, the present work aimed to examine baseline levels of accurate and biased perceptions of gratitude, and their unique contributions to relationship satisfaction, an important indicator of relationship well-being (Rusbult, 1983). Dissecting perceptions into accuracy and bias using advanced statistical methods paints a fuller picture of how gratitude may promote positive social outcomes for both members of the dyad, provides greater nuance to previous findings and informs future intervention work regarding what component(s) on which to focus.

## Accuracy and Bias as Independent Constructs

Accuracy and bias can be independent or correlated depending on various factors (Fletcher & Kerr, 2010; Gagné & Lydon, 2004). To illustrate this idea, imagine Percy (Perceiver 1), Pete (Perceiver 2), and Penny (Perceiver 3) who perceive their partners’ gratitude levels as being 4, 5 and 6, respectively, on a 7-point scale. Their partners, Paris (Partner 1), Pam (Partner 2), and Paul (Partner 3), report on their own gratitude levels as 5, 6, and 7, respectively. In this example, perceivers know the relative levels of their partners’ gratitude, as the correlation between perceivers’ impressions and their partners’ self-reports is 1, indicating high tracking accuracy. But perceivers also underestimate the partners’ actual reports by 1 point, which is captured by a mean-level bias. As such, both tracking accuracy and bias can coexist, and other combinations of ratings may reveal different patterns (e.g., low-tracking accuracy and overestimation).

Are people accurate and/or biased when perceiving their partner’s gratitude? One study has shown that, when it comes to perceiving their partner’s daily expressions of gratitude, perceivers tracked daily fluctuations in their partner’s gratitude expressions, and they did not display a mean-level bias (Park, Impett, et al., 2019). While Park, Impett et al. (2019) examined daily fluctuations, tapping into more concrete and recent perceptions, the present research focuses on partners’ general gratitude, tapping into more global perceptions. Nevertheless, there is initial evidence that people are capable of accurately perceiving their partner’s gratitude. Although there was no mean-level bias when perceiving daily gratitude, biases may more easily color broad-based global estimates, as these judgments capture a summary of different instances (e.g., Neff & Karney, 2005). This idea relates to research indicating that people use mental shortcuts for efficient judgment and decision-making (Kahneman & Tversky, 1972; Tversky & Kahneman, 1973, 1974) Furthermore, from the perspective of Error Management Theory (*EMT*: Haselton & Buss, 2000), which posits that, in contexts of uncertainty, people systematically err on the side of caution by making less costly errors in judgments, it is plausible that people may underperceive their partners’ global gratitude. When in doubt, underperceiving could serve as a relationship maintenance strategy as it could promote more pro-relationship behavior by the perceiver (Fletcher & Kerr, 2010), whereas overperceiving could result in complacency and low effort, putting the maintenance of the relationship at risk.

Beyond tracking accuracy and mean-level bias, people are susceptible to other types of biases, such as an assumed similarity bias, which involves perceiving one’s partner as being similar to the self, above and beyond actual similarity. Assumed similarity bias in other areas, such as relationship attributes and values, has been positively associated with relationship satisfaction (Lutz-Zois et al., 2006; Murray et al., 2002). Although past work has found that perceivers display significant levels of assumed similarity bias when judging their partner’s daily gratitude expressions (Park, Impett, et al., 2019), it is unclear how assumed similarity bias may operate at a global level and how it is associated with relationship satisfaction.

## Links With Romantic Satisfaction

Accuracy and bias may relate to relationship satisfaction for perceivers and their partners in different ways. First, partners may be more satisfied with the relationship when perceivers more accurately see their partners’ gratitude, which may lead partners to feel understood by perceivers. Indeed, accuracy in perceiving partners’ attributes is associated with greater felt understanding among partners (Lackenbauer et al., 2010; but also see Pollmann & Finkenauer, 2009), which, in turn, could increase partners’ satisfaction with the relationship (A. M. Gordon & Chen, 2016; Pollmann & Finkenauer, 2009). Therefore, greater accuracy by the perceiver may be linked with greater relationship satisfaction for their partners.

Second, underestimating a partner’s gratitude a great deal is likely associated with greater dissatisfaction compared with underestimating to a lesser degree or maybe even overestimating a partner’s gratitude. This is because believing a partner is more grateful for oneself may be a more rewarding experience in itself and may help perceivers feel good about their contributions to their partners’ personal and relational well-being (Park et al., 2020).

Finally, assumed similarity bias could also be linked to relationship satisfaction, especially for perceivers. This may be due to an egocentric process of projecting one’s level of gratitude onto the partner (e.g., Murray et al., 2002), resulting in the belief that the partner is equally grateful as the perceiver and that both members mutual contributors to the relationship, which has been previously linked to higher satisfaction (Sprecher et al., 2016). Thus, assumed similarity bias might relate to greater perceiver satisfaction by fostering a sense of mutuality.

## Research Overview

Employing two dyadic studies, one exploratory and one confirmatory, we examined accuracy and bias in perceiving a partner’s general gratitude and their contributions to relationship satisfaction. In Study 1, we explored the cross-sectional links between accuracy and bias in perceiving a partner’s *gratitude experience* and relationship satisfaction. In Study 2, we examined the cross-sectional links between accuracy and bias in perceiving a partner’s *gratitude expression* and relationship satisfaction across 2 time points (3 months apart), providing a within-study replication. We also examined the lagged effects between accuracy and bias and relationship satisfaction. In both studies, we also considered gender and relationship length as moderators. Due to space limitations, findings from the moderators for both studies and the lagged analyses from Study 2 appear in the Supplementary Online Materials (SOM). Overall, the pattern of results was consistent across genders and relationship lengths. The lagged analyses did not provide clear support for either directionality. Of note, for both studies, we report on all measures, data exclusions, and analyses conducted related to the present research. The study codebooks as well as the data and R code are available online: https://osf.io/tsxhw/?view_only=76d9ae91633d4792aa7f2963c7925a6d.

## Study 1

### Method

#### Sample

Romantic couples within the age of 18 to 35 and in a relationship with the same partner for at least 3 months were recruited from a university community. In total, 468 participants completed the study. Participants were compensated with one extra course credit or $10.00 in cash or via an amazon.ca e-gift card.

Participants were removed from analyses if only one partner from the couple participated in the study (*n* = 14). Participants were also excluded if they (*n* = 26) or their partner (*n =* 28) failed the attention check (see study codebook for details about the attention checks). The final sample consisted of 410 participants (*N*_dyads_ = 205; 52.4% women; 81.5% heterosexual; *M*_age_ = 20.99 years; *SD*_age_ = 2.68; *M*_relationshipLength_ = 23.13 months, *SD*_relationshipLength_ = 18.65).

The present sample size was determined based on general recommendations in the field (Fraley & Vazire, 2014; Vazire, 2014) indicating that a sample of 200 to 250 participants is sufficient to detect the average observed effect size in social psychology (*r* = .21; Fraley & Marks, 2007; Richard et al., 2003). The present study consists of a total of 410 participants (205 couples), which is expected to provide ample power to detect the average published effect size. We also conducted post hoc power analyses to estimate the achieved power. To do so, we first computed the effective sample size (*N*_eff_ = 333.66) by taking into account the dependency of the data (intraclass correlation [ICC] = .27). Power calculations using GPower (Faul et al., 2009) indicated that our sample provides ample power (97.4%) to detect an effect size of *r* = .21.

#### Procedure and Measures

Participants were emailed a survey and were instructed to complete it independently. Among other measures, participants reported on their own and their partner’s gratitude by indicating the extent to which they and their partner typically felt “appreciative,”“thankful,” and “grateful” when interacting with each other (Algoe et al., 2010). Their relationship satisfaction was assessed using the 3-item satisfaction subscale from the assessment of relationship commitment scale (Gagné & Lydon, 2003). See Table 1 for variable descriptives.

**Table 1:** Summary of Variable Descriptives and Correlations for Study 1

Variable	Descriptives				Correlations	
	Scale	*M*	*SD*	α	1	2
1. Self-reported gratitude	1 —*Almost never* to 5—*Almost always*	4.43	0.69	.91	—	
2. Perceived gratitude in partner		4.14	0.85	.95	.35	—
3. Relationship satisfaction	1—*Not at all* to 9—*Completely*	7.93	1.15	.87	.55	.56

*Note*. All correlations were significant at *p* < .05. *SD* = standard deviation.

#### Analytical Approach

Analyses were conducted using the *nlme* package (Pinheiro et al., 2022) for R (R Development Core Team, 2019). The data were structured such that individuals were nested within dyads. Following the guidelines of the Truth and Bias Model of Judgment (West & Kenny, 2011), we estimated a series of multilevel models, accounting for the dyadic interdependence. Because our data includes both mixed and same-gender dyads, following recent recommendations (Stern & West, 2017), we modeled random intercepts using a compound symmetry structure.

In the baseline model, we predicted perceivers’ judgments of partners’ gratitude experience from partners’ self-reported gratitude and perceivers’ self-reported gratitude. All variables were centered on the mean of partners’ gratitude experience. All predictors were standardized prior to analyses for consistency across studies. The data and the R code for conducting these analyses with unstandardized predictors are available at https://osf.io/tsxhw/?view_only=76d9ae91633d4792aa7f2963c7925a6d. The pattern of results obtained with unstandardized predictors is the same as those reported here.

The model intercept reflects the mean-level bias at the sample level. For example, a negative coefficient would indicate that perceivers generally underestimate their partners’ gratitude. An index of tracking accuracy was obtained by the regression coefficient indicating the association between partners’ own gratitude experience and perceivers’ judgments of partners’ gratitude, controlling for perceivers’ self-rated gratitude. Finally, assumed similarity was indexed by the regression coefficient indicating the association between perceivers’ reports of gratitude and perceivers’ judgments of partners’ gratitude, controlling for partners’ actual gratitude.

To examine the links with relationship satisfaction, we added standardized relationship satisfaction as a moderator (i.e., a predictor of the intercept and the accuracy and assumed similarity slopes). In doing so, we followed the approach used by Spielmann et al. (2020), although these authors did not use this approach to examine the links between mean-level bias and satisfaction (only for accuracy/assumed similarity and satisfaction). However, this method also reveals the association between mean-level bias and satisfaction (West & Kenny, 2011; also see Tissera & Lydon, 2021). As such, we simultaneously estimated the links between (a) mean-level bias and relationship satisfaction, (b) tracking accuracy and relationship satisfaction, and (c) assumed similarity bias and relationship satisfaction. Although this analytical approach involves including relationship satisfaction as a moderator, it is conceptualized as the outcome (Spielmann et al., 2020; Tissera & Lydon, 2021 also see Human et al., 2013, 2020; for a similar approach with the Social Accuracy Model). Separate models were estimated for perceiver and partner satisfaction.

### Results

All results are reported in Table 2.

**Table 2:** Summary of Results Across Both Studies

Perceptual component	Baseline levels				Links with perceiver satisfaction				Links with partner satisfaction			
	*B* [CI]	*SE*	*t*	*r*	B [CI]	*SE*	*t*	*r*	B [CI]	*SE*	*t*	*r*
Study 1												
Tracking accuracy	0.23** [0.15, 0.30]	0.04	6.25	.29	−0.04 [−0.10, 0.03]	0.03	−1.13	−.05	0.08** [0.03, 0.16]	0.03	3.17	.15
Mean level bias	−0.29** [-0.36, -0.22]	0.04	−7.92	−.36	0.18** [0.08, 0.27]	0.05	3.77	.18	0.07 [−0.02, 0.17]	0.05	1.61	.08
Assumed similarity bias	0.33** [0.25, 0.40]	0.04	8.99	.40	0.07** [0.02, 0.12]	0.03	2.74	.13	−0.04 [−0.11, 0.03]	0.03	−1.31	−.06
Study 2												
Time 1 (Baseline)												
Tracking accuracy	0.49** [0.38, 0.60]	0.06	8.73	.33	−0.12* [−0.22, −0.02]	0.05	−2.45	−.10	−0.02 [−0.12, 0.07]	0.05	−0.49	−.02
Mean level bias	−0.19** [−0.29, −0.08]	0.05	−3.50	−.13	0.70** [0.58, 0.82]	0.06	11.32	.41	0.29** [0.16, 0.42]	0.07	4.39	.17
Assumed similarity bias	0.48** [0.37, 0.59]	0.06	8.49	.32	0.20** [0.11, 0.28]	0.04	4.52	.18	−0.02 [-0.12, 0.09]	0.05	−0.31	−.01
Time 2 (3 months later)												
Tracking accuracy	0.47** [0.35, 0.61]	0.07	7.28	.34	0.01 [−0.10, 0.11]	0.05	0.12	.01	0.04 [−0.09, 0.29]	0.05	0.77	.04
Mean level bias	−0.13* [-0.25, -0.01]	0.06	−2.13	−.11	0.77** [0.62, 0.92]	0.08	10.14	.45	0.40** [0.23, 0.56]	0.08	4.84	.24
Assumed similarity bias	0.63** [0.50, 0.76]	0.07	9.53	.43	0.26** [0.16, 0.36]	0.05	5.33	.26	0.07 [−0.04, 0.18]	0.06	1.14	.06

*Note. B* = estimate, CI = 95% confident interval calculated using parametric bootstrapping method with 1000 simulations, *SE =* standard error, effect sizes (*r*) were obtained using the formula from Rosenthal and Rosnow (2008): *r* = $\sqrt{\frac{t^{2}}{t^{2}+\mathrm{df}}}$ .

** *p* < .01. * p < .05.

#### Baseline Levels

Perceivers accurately judged the relative levels of their partners’ gratitude, evidenced by significant levels of tracking accuracy. Furthermore, perceivers underestimated their partners’ gratitude as indicated by a significant negative mean-level bias. Finally, perceivers relied on their own level of gratitude when inferring their partners’ gratitude experience as indicated by a significant assumed similarity bias.

#### Links with Relationship Satisfaction

##### Perceivers

Tracking accuracy was not significantly related to perceiver relationship satisfaction. However, mean-level bias was significantly and positively associated with perceivers’ relationship satisfaction. Given that perceivers, on average, underestimated their partners’ gratitude experience, we interpret this association as perceivers being less satisfied when they underestimate their partners’ gratitude to a greater degree. Furthermore, greater assumed similarity bias was significantly related to perceivers’ relationship satisfaction. That is, perceivers who believed their partners experienced similar levels of gratitude as they did were more satisfied.

##### Partners

Greater tracking accuracy was significantly related to greater relationship satisfaction for partners such that partners were more satisfied when perceivers more accurately gauged the relative levels of their partners’ gratitude. Partners’ relationship satisfaction was not significantly related to mean-level bias or assumed similarity bias.

## Study 2

In Study 2, we aimed to provide a preregistered replication and extension of the results from Study 1 by examining perceptions of a partner’s gratitude expression. Furthermore, leveraging the longitudinal nature of this study (2 time points 3 months apart), we provide a within-sample replication of the links explored in Study 1. We preregistered all analyses (https://osf.io/ynekx/?view_only=7fea8c73f3c947b288882418850cb1ad).

### Method

#### Sample

The present study employs Integrated Data Analyses (IDA: Curran & Hussong, 2009; Hussong et al., 2013), which is an innovative analytical approach that allows the analysis of raw data pooled across multiple samples. The data for the present research were drawn from three independent, longitudinal, dyadic samples (referred to as Samples 2a–2c). The final sample consisted of 634 participants (50.6% women; *M*_age_=29.07, *SD*_age_=9.41; *M*_relationshipLength_=5.57, *SD*_relationship length_=4.83). Details about each of the individual samples appear in the preregistration.

Although it was not preregistered, we conducted power analyses based on the smallest observed effect from Study 1, which is the association between assumed similarity bias and perceiver satisfaction (*r = .*13). Power was estimated using GPower (Faul et al., 2009). Having taken into account the dependence of the data (ICC = .30; *N*_effective_ = 486.65), we found that the present sample is adequately powered (82.5%) for the present analyses.

#### Procedure

Participants completed a background survey and were contacted again 3 months later (*N* = 502). At both time points, participants reported their relationship satisfaction, self- and partner-reports of gratitude expression, and other well-being measures for purposes beyond this project.

#### Measures

##### Gratitude

Self- and partner-reports of gratitude expression were each indexed using 1 item from the Appreciation in Relationships Scale (Gordon et al., 2012; also see Park, Impett, et al., 2019). Specifically, using a 7-point Likert-type scale (1 = *Strongly Disagree*, 7 = *Strongly Agree*), participants rated the items “I make sure my partner feels appreciated” and “My partner makes sure I feel appreciated.”

##### Relationship Satisfaction

In Samples 2a and 2b, relationship satisfaction was indexed using the 3-item subscale from the Perceived Relationship Quality Components inventory (Fletcher et al., 2000; *M_Time1_* = 5.88, *SD_Time1_*=1.09; α _Time1_=0.94; *M_Time2_* = 5.81, *SD_Time2_* = 1.31; α _Time2_ = 0.98). Items were rated on a 7-point Likert-type scale (1 *= Not at All*, 7 *= Extremely)*. In Sample 2c, relationship satisfaction was indexed using the relationship satisfaction subscale from the Investment Model Scale (Rusbult et al., 1998; *M_Time1_* = 5.86, *SD_Time1_* = 1.19, α _Time1_ = 0.94; *M_Time2_* = 5.76, *SD_Time2_* = 1.25; α _Time2_ = 0.95). Participants reported on 5 items using a 7-point Likert-type scale (1 *= Strongly Disagree*, 7 *= Strongly Agree).* Although relationship satisfaction was measured using two different scales across studies, we believe that both measures were indexing the same underlying construct, and as such, IDA is deemed appropriate (for a similar approach employing the same measures see Overall, 2020; Sasaki & Overall, 2020). Before analyses, we standardized relationship satisfaction within the sample, removing sample level mean and variance differences (see Rodriguez et al., 2018 for a similar approach). See Table 3 for the overall descriptives and correlations of relationship satisfaction.

**Table 3:** Summary of Variable Descriptives and Correlations for Study 2

Variable	Descriptives		Correlations				
	*M*	*SD*	1	2	3	4	5
Self-reported gratitude (Time 1)	5.50	1.23	—				
Perceived gratitude in partner (Time 1)	5.31	1.53	.41	—			
Relationship satisfaction (Time 1)	5.88	1.13	.50	.59	—		
Self-reported gratitude (Time 2)	5.45	1.31	.53	.34	.38	—	
Perceived gratitude in partner (Time 2)	5.27	1.54	.33	.46	.42	.54	—
Relationship satisfaction (Time 2)	5.81	1.28	.33	.41	.60	.59	.62

*Note*. All correlations were significant at *p* < .05. *SD* = standard deviation.

#### Analytical Approach

The present analyses employed IDA (Curran & Hussong, 2009), a method of data pooling that maximizes statistical power, increases sample heterogeneity, and provides reliable estimates of effects across studies. Because this study only included three samples, we employed a fixed-effects IDA as opposed to a random-effects IDA which is recommended for pooling across a large number of datasets. The fixed-effects method involves controlling for the sample as an additional predictor in the model. Separate analyses were conducted for the Time 1 concurrent data and the Time 2 concurrent data to obtain a within-sample replication. Beyond the above-mentioned changes, all cross-sectional analyses in Study 2 were identical to the procedures outlined in Study 1 above and are also detailed in the preregistration.

### Results

All results are reported in Table 2.

#### Baseline Levels

Perceivers displayed significant levels of tracking accuracy, indicating that perceivers knew their partners’ relative level of gratitude. Furthermore, perceivers displayed a significant negative mean-level bias, suggesting that perceivers generally underestimated their partners’ gratitude. Perceivers also displayed a significant assumed similarity bias, which indicates that they believed their partners’ gratitude was similar to their own. This pattern of results was consistent across both time points assessed in this study and with the findings from Study 1.

#### Links with Relationship Satisfaction

##### Perceivers

We found that Time 1 tracking accuracy was significantly negatively associated with perceiver relationship satisfaction, suggesting that knowing their partners’ relative levels of gratitude was associated with lower satisfaction for perceivers. However, this link did not replicate at Time 2 and was not consistent with the findings from Study 1. Moreover, as in Study 1, concurrent mean-level bias and assumed similarity bias were significantly and positively related to perceiver relationship satisfaction at both time points. This suggests that perceivers were more satisfied with their relationships when they perceived greater gratitude from their partner. Given that perceivers generally underestimated their partners’ gratitude, this finding can be interpreted as greater underestimation being associated with lower perceiver satisfaction. In addition, believing the partner was expressing a similar level of gratitude as the perceiver was also related to perceiver relationship satisfaction.

##### Partners

In terms of partners’ concurrent relationship satisfaction, the findings for Study 2 deviated from those of Study 1. Partners’ satisfaction at both time points was significantly and positively associated with mean-level bias but not with tracking accuracy. As in Study 1, there were no associations between assumed similarity bias and partners’ relationship satisfaction. Thus, in Study 2, partners reported being more satisfied with their relationship when the perceivers underestimated their gratitude to a lesser extent.

## Discussion

Thus far, little work has examined whether people are accurate or biased when perceiving a partner’s gratitude (Park, Impett, et al., 2019), and no research has examined how these perceptual components are associated with relationship well-being. Across two dyadic studies, the present work decomposed people’s perceptions of their romantic partners’ gratitude experience and expression into accuracy and bias and examined their roles in relationship satisfaction.

### Tracking Accuracy

People accurately perceived the relative levels of their partner’s gratitude experience and expression, evidenced by significant levels of tracking accuracy at baseline, consistent with findings from Park, Impett, et al. (2019) regarding specific instances of partner gratitude. Could tracking accuracy contribute to relationship satisfaction? It appears that perceivers’ satisfaction may not benefit from tracking accuracy. However, for partners, satisfaction was associated with accuracy in Study 1, perhaps signaling a sense of understanding to the partner. In turn, partners, feeling understood by perceivers, may report being more satisfied with their relationship (A. M. Gordon & Chen, 2016; Pollmann & Finkenauer, 2009). However, this link did not replicate in Study 2, which could be because this effect is unique to gratitude experience and does not translate to gratitude expression. The latter may not always be authentic and may be motivated by other factors, such as social norms or manipulative attempts to influence the partner. Therefore, partners’ relationship satisfaction may not benefit from partners having their gratitude expressions accurately perceived—but only from having their internal grateful experience accurately read. Nonetheless, future research is needed to replicate this finding to establish its reliability. Of note, partners’ satisfaction was also negatively related to tracking accuracy of gratitude expression in Study 2, but this did not replicate at the second time point. Thus, it is unclear how reliable this finding is, and we are hesitant to interpret it.

### Mean-Level Bias

We found that people generally underestimated their partner’s gratitude, in terms of both experience and expression. These results deviate from the findings by Park, Impett, et al. (2019), as they did not observe any mean-level bias when perceiving a partner’s daily gratitude expression. Aside from the Park, Impett, et al. (2019) findings being based on a smaller sample size (*N*_dyads_ = 78), we also hypothesized that the present work’s focus on global perceptions of a partner’s gratitude (vs. specific daily instances) would invite greater error of judgment and leave more room for biases to color perceptions of a partner’s gratitude.

Underestimating a partner’s general gratitude could serve as a relationship maintenance strategy, supporting the EMT (Haselton & Buss, 2000). Whenever there is uncertainty and the costs associated with making certain errors (i.e., under/overestimating) are asymmetric, people tend to be biased in the less costly direction. Indeed, underestimating a partner’s gratitude would be less costly than overestimation, which might involve taking the partner’s positive regard for granted (Fletcher & Kerr, 2010). Having a modest impression of the partner’s gratitude toward the self may motivate romantic partners to continue investing in and working on their relationship. Thus, underestimation of a partner’s general gratitude aligns with EMT (Haselton & Buss, 2000). Furthermore, this may be unique to general levels of gratitude (as opposed to concrete perceptions of gratitude) because people rate their general evaluations of their relationship and partner as being more important for the relationship than specific evaluations (Neff & Karney, 2002). This may be one reason why Park, Impett, et al. (2019) did not observe any directional bias for concrete gratitude perceptions, further underscoring the importance of examining gratitude perceptions at both global and specific levels.

Mean-level bias was related to relationship satisfaction. For perceivers, larger underestimations of their partners’ gratitude, implying perceiving lower partner gratitude, were related to lower satisfaction in both studies. Partners also reported lower relationship satisfaction the more the perceivers underestimated their gratitude expression, but not experience. Underestimating a partner’s gratitude may indicate that perceivers do not understand their partners and see them in a more negative light. Given that understanding and positive biases are key ingredients of relationship satisfaction (A. M. Gordon & Chen, 2016; Murray et al., 1996; Murray & Holmes, 1997; Pollmann & Finkenauer, 2009), low levels of each may produce a recipe for dissatisfaction. This goes hand-in-hand with the idea that having one’s gratitude perceived as being high may be rewarding for partners, as people typically tend to enjoy positive illusions (e.g., Murray et al., 1996; Murray & Holmes, 1997). In line with EMT, the association between mean-level bias and lower satisfaction also adds to the notion that modest underestimations of gratitude could be adaptive (Haselton & Buss, 2000). That is, if underestimations are related to lower satisfaction (an affective evaluation of the relationship), it could indicate that the relationship needs work to be maintained and be satisfactory. This also aligns with the research indicating that ambivalent feelings toward a partner motivate relationship improvement (Faure et al., 2022).

Notably, the link between mean-level bias and partner satisfaction was only observed in Study 2; therefore, it is not clear whether these findings extend to gratitude experience. Future research is needed to better understand the processes underlying perceptions of gratitude experience versus expression.

### Assumed Similarity

Perceivers demonstrated an egocentric bias by assuming their partner’s gratitude was similar to their own, above and beyond the partner’s actual gratitude level, a finding consistent with Park, Impett, et al. (2019). Furthermore, assumed similarity bias was related to greater satisfaction for perceivers but not partners. That is, perceiving greater similarity with the partner’s gratitude level, above and beyond the partner’s actual gratitude, was related to greater relationship satisfaction for perceivers. Perhaps an assumed similarity bias fosters a sense of mutuality in the relationship, signaling that both partners are equally grateful for each other. Because gratitude often arises when perceiving the benefits received from another’s actions, the mutuality explanation is consistent with research suggesting that believing both members are equal contributors to relationship maintenance is related to greater relationship satisfaction (Sprecher et al., 2016). That said, the present sample consisted of largely happy, grateful couples, making it unclear whether people will still value equality when projecting lower levels of gratitude, which should be empirically tested in the future.

### Directionality

Although the present work aimed to provide initial evidence of directionality, our lagged analyses did not reveal evidence for either direction of effects (see SOM). While accuracy and bias were linked to concurrent relationship satisfaction, perhaps other factors, such as relationship insecurities (e.g., Overall et al., 2012, 2015), may matter more in shaping accuracy and bias as well as relationship satisfaction over time. However, we are cautious in interpreting the null associations from our lagged analyses because it is possible that these analyses were underpowered. Although we had sufficient power to detect concurrent associations at both time points, our lagged analyses consisted of highly stringent tests and required estimating several two-way interactions within the same model. Future research is needed formally test the causality of these associations, ideally using experimental designs.

### Implications, Caveats, and Future Directions

It is important to underscore the broader implications of these findings. First, our results regarding a negative mean-level bias provide initial evidence that EMT generalizes to how people perceive others’ gratitude toward them. Second, aligning with past work (Gordon et al., 2011), our findings illuminate that experiencing and expressing gratitude have different interpersonal implications, a noteworthy distinction that is not to be overlooked. By examining the accuracy and bias of perceiving both the experience and the expression of gratitude, the present research paints a more complete picture of how perceptions of gratitude are associated with relationship satisfaction. Finally, the finding that people are both biased and accurate in perceiving their partner’s gratitude, and that gratitude accuracy and bias have independent implications for relationship satisfaction, contribute to the burgeoning literature on the interplay between accuracy and bias.

There are important limitations of the present research. For example, additional research is needed to establish the generalizability of these results as the current samples consisted of largely younger dating couples in heterosexual relationships. Future research is also needed to examine these associations in other types of populations (e.g., couples from varied sociocultural backgrounds) and relationships (e.g., colleagues).

In conclusion, perceivers significantly tracked their partners' general gratitude, while underestimating their partner's gratitude experience and expression and assuming their partner's gratitude was similar to their own. The biases in perceiving gratitude were related to perceivers' own relationship satisfaction. More precisely, perceivers seemed to benefit from assuming that their partners' gratitude was similar to their own but perceivers seemed to hurt from the underestimation of their partners' gratitude. Additionally, partners' relationship satisfaction appeared to benefit from perceivers holding more accurate impressions of partners' gratitude experience (but not expression) and appeared to hurt from perceivers underperceiving their gratitude expression (but not experience). We highlight that underperceiving partners' gratitude appears to be related to lower relationship satisfaction for both members of the couple, although this link was limited to gratitude expression for partners. Overall, this work adds to the growing body of literature on the interpersonally adaptive nature of gratitude by unraveling the accuracy and bias in perceiving others' gratitude levels and their unique links with relationship well-being.

## Supplemental Material

sj-docx-1-spp-10.1177_19485506221137958 – Supplemental material for Understanding the Links Between Perceiving Gratitude and Romantic Relationship Satisfaction Using an Accuracy and Bias Framework Click here for additional data file. Supplemental material, sj-docx-1-spp-10.1177_19485506221137958 for Understanding the Links Between Perceiving Gratitude and Romantic Relationship Satisfaction Using an Accuracy and Bias Framework by Hasagani Tissera, Mariko L. Visserman, Emily A. Impett, Amy Muise and John E. Lydon in Social Psychological and Personality Science
