# Supplementary material for: Understanding the Links Between Perceiving Gratitude and Romantic Relationship Satisfaction Using an Accuracy and Bias Framework
Source: Soc Psychol Personal Sci. 2022 Dec 2;14(8):900–10. doi: 10.1177/19485506221137958 (PMC10516740; doi:10.1177/19485506221137958)
Supplement: sj-docx-1-spp-10.1177_19485506221137958 – Supplemental material for Understanding the Links Between Perceiving Gratitude and Romantic Relationship Satisfaction Using an Accuracy and Bias Framework [file sj-docx-1-spp-10.1177_19485506221137958.docx]

**Understanding the Links Between Perceiving Gratitude and Romantic Relationship Satisfaction Using an Accuracy and Bias Framework**

**Supplementary Online Materials**

**Moderators**

For both studies, as per our preregistration, we considered both gender and relationship length as potential predictors of baseline levels of accuracy and bias, as well as moderators of their links with relationship satisfaction. The analytical approach for these analyses was highly similar to the approach explained in the main text. First, to examine whether gender predicted baseline levels of accuracy and bias, we added it as a predictor in the model, and as a moderator of tracking accuracy and assumed similarity bias, paralleling the analyses with relationship satisfaction outlined in the main text. Second, to examine whether gender moderated the links between accuracy and bias and relationship satisfaction, we added gender as a moderator of the link between relationship satisfaction and mean-level bias, relationship satisfaction and tracking accuracy slope, and relationship satisfaction and assumed similarity bias slope. The R script and the data necessary to replicate these analyses are available online on Open Science Framework: https://osf.io/tsxhw/?view_only=76d9ae91633d4792aa7f2963c7925a6d. All results for gender are summarized in Tables S1 and S2 below. We also conducted another set of analyses with relationship length as the moderator, which is summarized in Table S3. Of note, for analyses involving gender, we excluded participants who did not disclose their gender (*n*_study1_ = 1, *n*_study2_ = 1).

**Table S1**

*Baseline Accuracy and Bias Moderated by Perceiver Gender*

|  | *Moderation Effect* | | | *Simple Slope for Women* | | | *Simple slope for Men* | | |
| --- | --- | --- | --- | --- | --- | --- | --- | --- | --- |
| Perceptual Component | *b* | *SE* | *t* | *b* | *SE* | *t* | *b* | *SE* | *t* |
| Study 1 |  |  |  |  |  |  |  |  |  |
| Tracking accuracy | 0.05 | 0.07 | 0.69 | 0.24** | 0.05 | 4.75 | 0.19** | 0.05 | 3.69 |
| Mean level bias | -0.17* | 0.07 | -2.43 | -0.37** | 0.05 | -7.41 | -0.20** | 0.05 | -3.75 |
| Assumed similarity bias | 0.04 | 0.07 | 0.58 | 0.35** | 0.05 | 7.06 | 0.31** | 0.05 | 5.84 |
| Study 2 |  |  |  |  |  |  |  |  |  |
| *Time 1* |  |  |  |  |  |  |  |  |  |
| Tracking accuracy | -0.45** | 0.11 | -4.05 | 0.70** | 0.08 | 8.94 | 0.25** | 0.08 | 3.19 |
| Mean level bias | 0.30** | 0.10 | 3.00 | -0.31** | 0.07 | -4.29 | -0.01 | 0.07 | -0.11 |
| Assumed similarity bias | 0.19† | 0.11 | 1.74 | 0.39** | 0.08 | 4.84 | 0.58** | 0.08 | 7.61 |
| *Time 2* |  |  |  |  |  |  |  |  |  |
| Tracking accuracy | -0.43** | 0.13 | -3.28 | 0.72** | 0.10 | 7.38 | 0.28** | 0.09 | 3.22 |
| Mean level bias | 0.11 | 0.12 | 0.95 | -0.18* | 0.08 | -2.14 | -0.07 | 0.09 | -0.80 |
| Assumed similarity bias | 0.03 | 0.13 | 0.26 | 0.61** | 0.09 | 6.99 | 0.64** | 0.10 | 6.42 |

*Note.* ** *p* < .01, *** *p* < .05, † < .10

Overall, it appears that women were better at tracking their partners’ gratitude expressions than men. Although we observed a similar pattern for tracking accuracy in Study 1, the moderation effect was not statistically significant. Previous work on empathic accuracy has also found similar gender differences, but this has been attributed to differences in motivation (e.g., communal orientation), rather than ability (Ickes et al., 2000; Klein & Hodges, 2001). As such, these different motivations may also be driving the gender effects observed here, although more rigorous research geared towards this specific question is needed to confirm this hypothesis.

We also found that women tended to underestimate their partner’s gratitude, more so than men. One possibility is that women may be more communally oriented, which may be driving this effect. To the extent that underestimation is adaptive for relationship maintenance (as discussed in the main manuscript), women may engage in greater underestimation. However, this moderation effect was not significant at Time 2 of Study 2. Given the mixed findings, it is unclear how reliable this finding is.

**Table S2**

*Links with Concurrent Relationship Satisfaction Moderated by Perceiver Gender*

|  | *Moderation Effect for Partner Satisfaction* | | | *Moderation Effect for Perceiver Satisfaction* | | |
| --- | --- | --- | --- | --- | --- | --- |
| Perceptual Component | *b* | *SE* | *t* | *b* | *SE* | *t* |
| Study 1 |  |  |  |  |  |  |
| Tracking accuracy | 0.08 | 0.6 | 1.35 | -0.02 | 0.07 | -0.30 |
| Mean level bias | 0.05 | 0.10 | 0.54 | 0.05 | 0.09 | -0.53 |
| Assumed similarity bias | 0.04 | 0.07 | 0.61 | 0.11† | 0.06 | 1.83 |
| Study 2 |  |  |  |  |  |  |
| *Time 1* |  |  |  |  |  |  |
| Tracking accuracy | -0.11 | 0.10 | -1.15 | -0.0001 | 0.10 | -0.001 |
| Mean level bias | -0.17 | 0.14 | -1.27 | -0.06 | 0.13 | -0.47 |
| Assumed similarity bias | 0.15 | 0.11 | 1.32 | -0.09 | 0.09 | -0.95 |
| *Time 2* |  |  |  |  |  |  |
| Tracking accuracy | 0.17 | 0.10 | 1.61 | 0.10 | 0.10 | 1.02 |
| Mean level bias | 0.15 | 0.17 | 0.90 | -0.56** | 0.15 | -3.62 |
| Assumed similarity bias | -0.11 | 0.11 | -0.94 | -0.17† | 0.10 | -1.75 |

*Note.* ** *p* < .01, † *p* < .10

Overall, gender did not significantly moderate any of the links with relationship satisfaction in Study 1. In Study 2, gender significantly moderated the association between mean-level bias and perceiver satisfaction at Time 2 only. When probing the simple slopes for this effect, we found that the association between relationship satisfaction and mean-level at Time 2 was stronger for women (*b* = 1.13, *t* = 9.82, *p* < .001) than men (*b* = 0.57, *t* = 5.63, *p* < .001), although for both genders, this link was significant and positive. Given we did not observe a similar link at Time 1, further research is needed to establish its reliability. Taken together, gender did not appear to moderate the links between accuracy and bias and relationship satisfaction in a consistent way.

**Table S3**

*Baseline Accuracy and Bias Moderated by Relationship Length*

|  | *Effect of Relationship Length at Baseline* | | | *Moderation Effect for Partner Satisfaction* | | | *Moderation Effect for Perceiver Satisfaction* | | |
| --- | --- | --- | --- | --- | --- | --- | --- | --- | --- |
| Perceptual Component | *b* | *SE* | *t* | *b* | *SE* | *t* | *b* | *SE* | *t* |
| Study 1 |  |  |  |  |  |  |  |  |  |
| Tracking accuracy | 0.01 | 0.03 | 0.23 | -0.001 | 0.02 | -0.08 | -0.03 | 0.04 | -0.77 |
| Mean level bias | -0.003 | 0.04 | -0.09 | -0.04 | 0.04 | -0.89 | 0.08† | 0.04 | 1.71 |
| Assumed similarity bias | -0.06* | 0.03 | -2.08 | -0.01 | 0.04 | -0.33 | -0.004 | 0.02 | -0.24 |
| Study 2 |  |  |  |  |  |  |  |  |  |
| *Time 1* |  |  |  |  |  |  |  |  |  |
| Tracking accuracy | 0.04 | 0.06 | 0.66 | 0.07 | 0.05 | 1.42 | -0.02 | 0.05 | -0.34 |
| Mean level bias | 0.002 | 0.06 | 0.04 | 0.01 | 0.08 | 0.16 | 0.01 | 0.07 | 0.17 |
| Assumed similarity bias | 0.05 | 0.06 | 0.89 | -0.02 | 0.05 | -0.48 | 0.02 | 0.04 | 0.44 |
| *Time 2* |  |  |  |  |  |  |  |  |  |
| Tracking accuracy | 0.05 | 0.06 | 0.76 | 0.13* | 0.06 | 2.24 | 0.09† | 0.05 | 1.75 |
| Mean level bias | -0.002 | 0.06 | -0.04 | 0.23** | 0.08 | 2.79 | -0.08 | 0.08 | -1.05 |
| Assumed similarity bias | -0.09 | 0.06 | -1.46 | -0.10† | 0.05 | -1.86 | -0.12* | 0.05 | -2.46 |

*Note.* ** *p* < .01, *** *p* < .05, †*p* < .10

Relationship length did consistently not moderate the baseline levels of accuracy and bias across both studies, although assumed similarity bias in Study 1 was stronger for those in shorter relationships (1 standard deviation below the mean) (*b* = 0.40, *t* = 7.95, *p <* .001), than longer relationships (1 standard deviation above the mean) (*b* = 0.28, *t* = 6.32, *p* < .001). We also did not observe any significant moderations by relationship length for the links between accuracy and bias and satisfaction in Study 1 and at Time 1 in Study 2. However, we did observe that the link between mean-level bias and partner satisfaction at Time 2 in Study 2 was not significant for those in shorter relationships (*b* = 0.09, *t* = 0.73, *p* = .465) and highly significant for those in longer relationships (*b* = 0.55, *t* = 4.93, *p* < .001). Relationship length also appeared to moderate the link between tracking accuracy and partners’ relationship satisfaction at Time 2, such that this link was not significant for those in shorter (*b* = -0.04, *t* = -0.37, *p* = .709), but it was positive and significant for those in longer relationships (*b* = 0.21, *t* = 3.00, *p* = .003). Finally, the link between assumed similarity bias and perceiver satisfaction appeared to also depend on the length of the relationship, such that it was only positively associated with relationship satisfaction for those in shorter relationships (*b* = 0.36, *t* = 4.41, *p* < .001), and not for those in longer relationships (*b* = -0.02, *t* = -0.32, *p* = .751). Because these moderation effects were not consistent across studies and time points, we are hesitant to interpret them. In general, the links between accuracy and bias and satisfaction did not dependent on the length of the relationship in a consistent manner.

**Lagged Analyses**

Leveraging the longitudinal nature of Study 2, we attempted to better understand the directionality of the associations between accuracy and bias and relationship satisfaction. To test lagged effects, we adopted the method used by Human et al. (2020). Specifically, we first examined whether mean-level bias, tracking accuracy, and assumed similarity bias at Time 1 were related to perceiver and partner satisfaction at Time 2 when controlling for Time 1 satisfaction. Here, we included both Time 1 and Time 2 satisfaction as predictors in the model, and as moderators of tracking accuracy and assumed similarity bias from Time 1. This allowed us to estimate whether mean-level bias at Time 1 was associated with relationship satisfaction at Time 2, controlling for relationship satisfaction at Time 1. Similarly, within the same model, we also examined the associations between tracking accuracy and assumed similarity bias at Time 1 and relationship satisfaction at Time 2, controlling for relationship satisfaction at Time 1.

We also explored the possibility of relationship satisfaction at Time 1 being a predictor of accuracy and bias in perceiving partner’s gratitude expression at Time 2. For this, building on the Time 2 baseline model (as described in the main text), we included both Time 1 and Time 2 relationship satisfaction as predictors and moderators of tracking accuracy and assumed similarity. This allowed us to control for the concurrent associations between accuracy and bias and relationship satisfaction at Time 2 to obtain a cleaner estimate of the lagged effects of Time 1 relationship satisfaction. Due to the modelling approach employed for indexing accuracy and bias simultaneously, we are unable to control for the accuracy and bias at Time 1. Nevertheless, past work employing similar approaches has successfully examined lagged effects using the approach described here (Human et al., 2020). For a more detailed description and the equations representing the statistical model, see the preregistration. In addition, data and R code for re-creating all primary analyses are available on Open Science Framework. The results for the lagged analyses are summarized in Table S4 and Table S5.

**Table S4**

*Time 1 Accuracy and Bias Predicting Time 2 Relationship Satisfaction, Controlling for Time 1 Satisfaction*

| Perceptual Component at Time 1 | *Partner Satisfaction at Time 2* | | | *Perceiver Satisfaction at Time 2* | | |
| --- | --- | --- | --- | --- | --- | --- |
|  | *b* | *SE* | *t* | *b* | *SE* | *t* |
| Tracking accuracy | 0.12 | 0.07 | 1.53 | -0.08 | 0.07 | -1.04 |
| Mean level bias | 0.13† | 0.08 | 1.69 | 0.11† | 0.07 | 1.72 |
| Assumed similarity bias | -0.07 | 0.08 | -0.80 | 0.11 | 0.07 | 1.62 |

*Note.* †*p* <.10

**Table S5**

*Time 1 Relationship Satisfaction Predicting Time 2 Accuracy and Bias, Controlling for Time 2 Satisfaction*

| Perceptual Component at Time 2 | *Partner Satisfaction at Time 2* | | | *Perceiver Satisfaction at Time 2* | | |
| --- | --- | --- | --- | --- | --- | --- |
|  | *b* | *SE* | *t* | *b* | *SE* | *t* |
| Tracking accuracy | 0.04 | 0.08 | 0.54 | -0.07 | 0.08 | -0.93 |
| Mean level bias | -0.05 | 0.08 | -0.58 | 0.05 | 0.07 | 0.73 |
| Assumed similarity bias | 0.03 | 0.09 | 0.29 | 0.14† | 0.07 | 1.90 |

*Note.* †*p* <.10

Overall, none of the lagged effects reached statistical significance. Given that these associations were not statistically significant, we are unable to draw any clear conclusions regarding their directionality. It is possible that accuracy and bias of gratitude perceptions do not *shape* relationship satisfaction over time and vice-versa. Perhaps there may be other more important proximal factors that might contribute to changes in relationship satisfaction and accuracy and bias over time. That said, it is important to acknowledge that these null effects could be attributed to the stringent nature of this test. For example, when examining the links between Time 1 accuracy and bias and Time 2 relationship satisfaction, we were not only controlling for Time 1 satisfaction, but we were also controlling for the associations between Time 1 satisfaction and accuracy and bias. This is because of the way we indexed accuracy and bias within the model. Accuracy and bias were not simple predictors in the model, rather they were indexed by the intercept and the slopes between variables in the model. Furthermore, although we conducted power analyses for our primary concurrent analyses (see main text), it is not clear whether the present sample provides sufficient power for detecting the longitudinal effects. The lagged effects require estimating several additional two-way interactions within the model, which may require more power. These effects may also be smaller than the concurrent effects, and consequently, may require greater power. Additionally, these constructs may have been rather stable over time, making it challenging to predict changes. For instance, satisfaction at Time 1 and Time 2 was correlated at *r =* .60 (see Table 3 in the main manuscript), and upon visual inspection, we note that the estimates for accuracy and bias are fairly similar at both time points (see Table 2 in the main manuscript). We encourage future research to further examine the causality of these links either by employing much larger samples or by using different methods, such as experimental paradigms.
